# Supplementary figures and images for: Recombinant expression of osmotin in barley improves stress resistance and food safety during adverse growing conditions
Source: PLoS One. 2019 May 10;14(5):e0212718. doi: 10.1371/journal.pone.0212718 (PMC6510477; doi:10.1371/journal.pone.0212718)

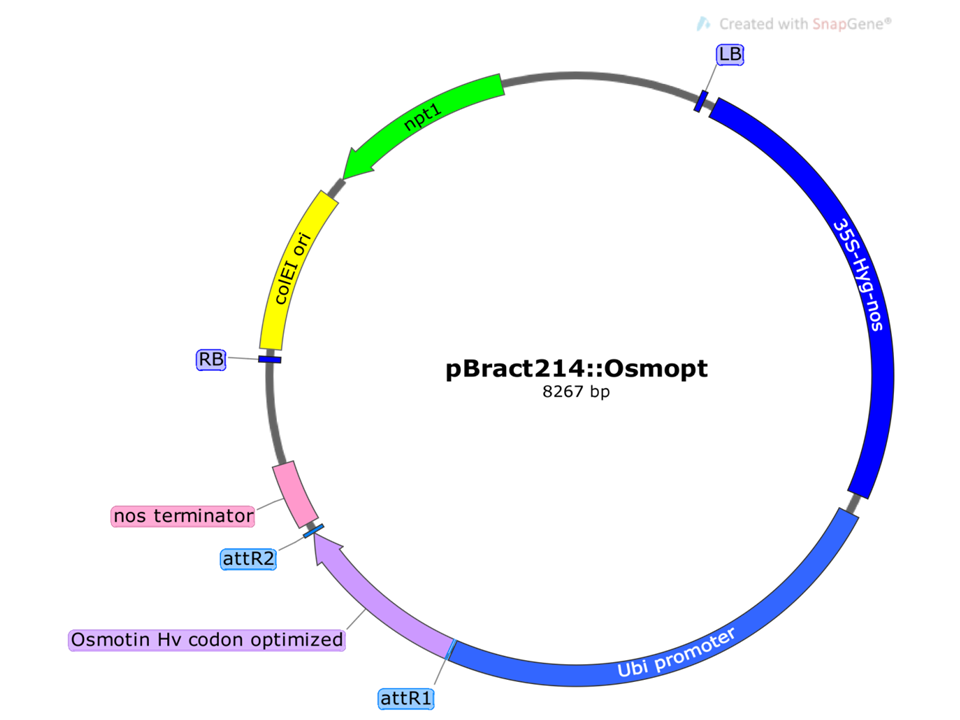

Supplement: S1 Fig — (TIF) [file pone.0212718.s001.tif]

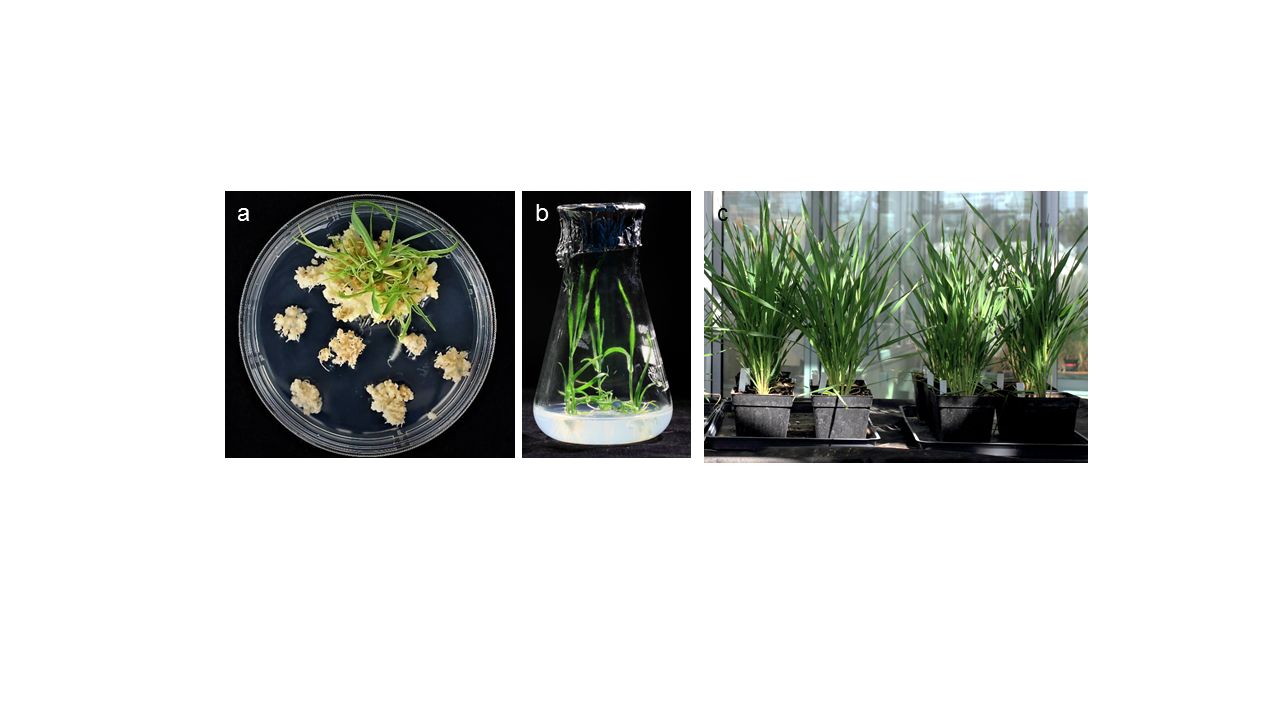

Supplement: S2 Fig — a) Regenerating plantlets from calluses after 6 wk on selection medium. b) Putative transgenic plants on regenerating medium. c) Transgenic plants T0 generation in greenhouse. (TIF) [file pone.0212718.s002.TIF]

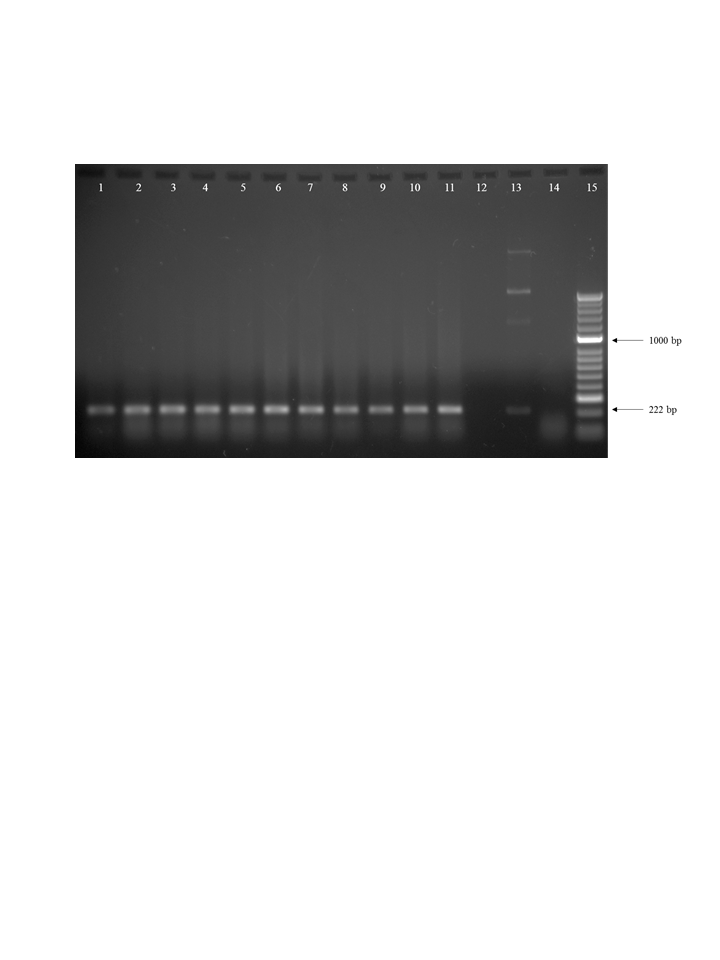

Supplement: S3 Fig — Lane 1–11: samples; lane 12: negative control (DNA/RNA free water); lane 13: positive control (plasmid pBRACT214::osm); lane 14: negative control (genomic DNA of WT plants); lane 15: DNA standard (50 bp DNA ladder, Bioline). Size of PCR product is 222 bp. (TIF) [file pone.0212718.s003.tif]

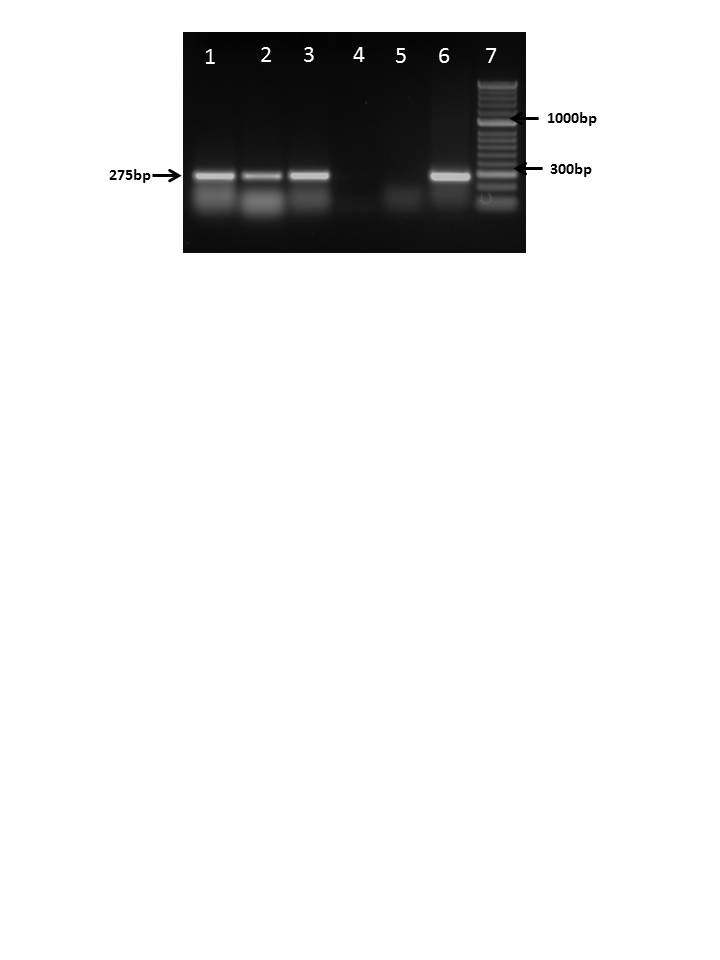

Supplement: S4 Fig — Lane 1–3: samples; lane 4: negative control (DNA/RNA free water); lane 5: negative control (genomic DNA of WT plants); lane 6: positive control (hpt positive plant), lane 7: DNA standard (2-kb DNA ladder, Bioline). Size of PCR product is 275 bp. (TIF) [file pone.0212718.s004.tif]

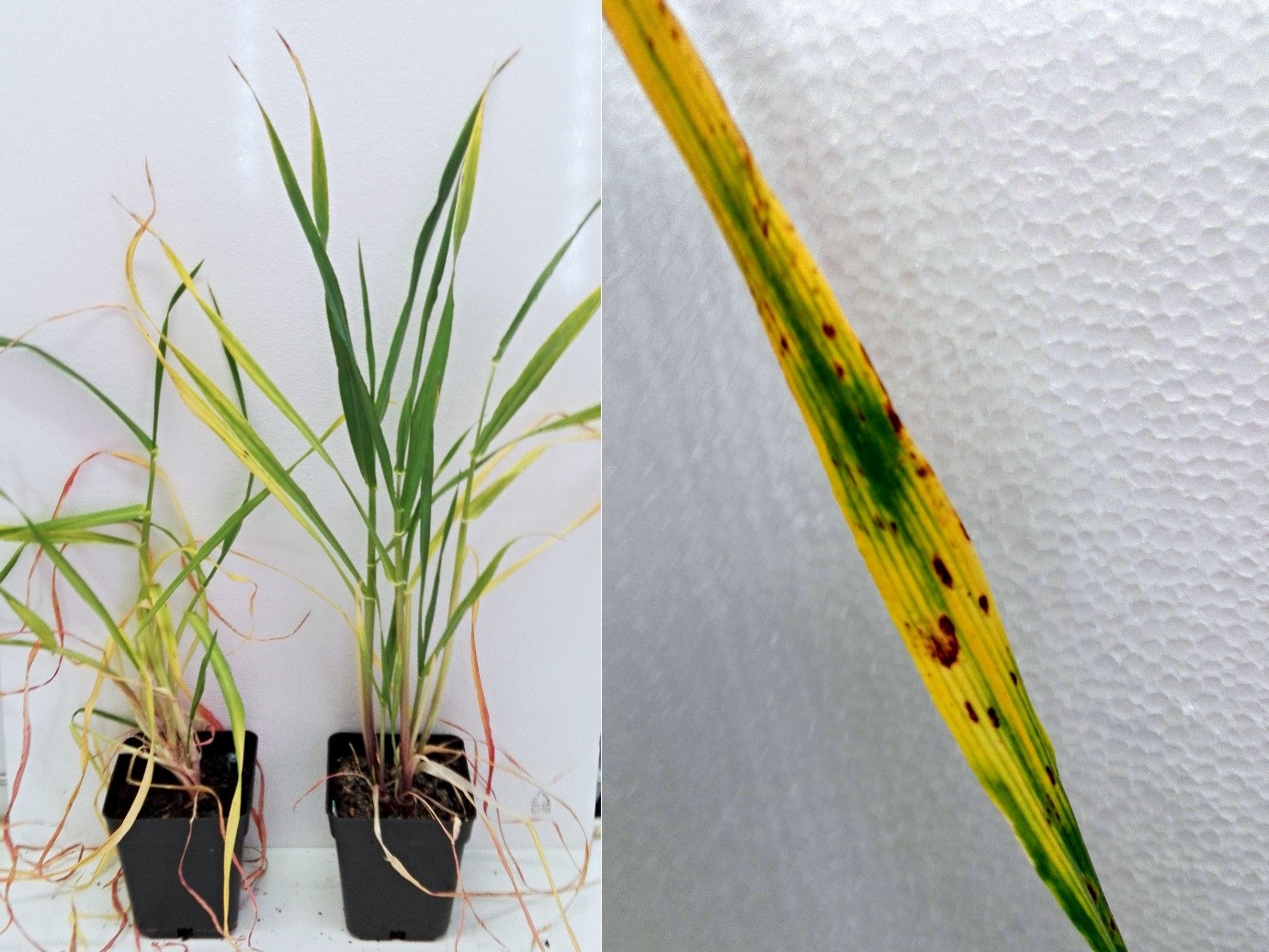

Supplement: S5 Fig — left: Non-transgenic barley (left) versus transgenic barley bearing tobacco osmotin gene (right) after biotic stress (15 days after first spraying of Fusarium oxysporum spores). Right: symptoms recognized on non-transgenic barley leaves after stress (chlorosis, necrosis, premature leaf drops and wilt of whole plant). (JPG) [file pone.0212718.s005.jpg]
